# Dendritic Cell Subpopulations Are Associated with Morphological Features of Breast Ductal Carcinoma In Situ

**DOI:** 10.3390/ijms24129918

**Published:** 2023-06-08

**Authors:** Joanna Szpor, Joanna Streb, Anna Glajcar, Anna Streb-Smoleń, Agnieszka Łazarczyk, Paulina Korta, Karolina Brzuszkiewicz, Robert Jach, Diana Hodorowicz-Zaniewska

**Affiliations:** 1 Department of Pathomorphology, Jagiellonian University Medical College, 31-008 Cracow, Poland; 2 Department of Pathomorphology, University Hospital, 30-688 Cracow, Poland; 3 Department of Oncology, Jagiellonian University Medical College, 31-008 Cracow, Poland; 4 Department of Oncology, Maria Sklodowska-Curie National Research Institute of Oncology, 31-115 Cracow, Poland; 5 General, Oncological, and Gastrointestinal Surgery, Jagiellonian University Medical College, 31-008 Cracow, Poland; 6 Department of Gynecology and Obstetrics, Jagiellonian University Medical College, 31-008 Cracow, Poland

**Keywords:** DCIS, dendritic cells, neoductgenesis, tumor microenvironment, CD1a, CD123, DC-LAMP, DC-SIGN

## Abstract

Ductal carcinoma in situ (DCIS) is the preinvasive form of breast cancer (BC). It is disputed whether all cases of DCIS require extensive treatment as the overall risk of progression to BC is estimated at 40%. Therefore, the crucial objective for researchers is to identify DCIS with significant risk of transformation into BC. Dendritic cells (DC) are professional antigen presenting cells and as such play a pivotal role in the formation of immune cells that infiltrate in breast tumors. The aim of this study was to investigate the relationship between the density of DCs with different superficial antigens (CD1a, CD123, DC-LAMP, DC-SIGN) and various histopathological characteristics of DCIS. Our evaluation indicated that CD123^+^ and DC-LAMP^+^ cells were strongly associated with maximal tumor size, grading and neoductgenesis. Together with CD1a^+^ cells, they were negatively correlated with hormonal receptors expression. Furthermore, the number of DC-LAMP^+^ cells was higher in DCIS with comedo necrosis, ductal spread, lobular cancerization as well as comedo-type tumors, while CD1a^+^ cells were abundant in cases with Paget disease. We concluded that different subpopulations of DCs relate to various characteristics of DCIS. Of the superficial DCs markers, DC-LAMP seems particularly promising as a target for further research in this area.

## 1. Introduction

Ductal carcinoma in situ (DCIS) is a noninvasive lesion that accounts for up to 90% of the precursors for invasive breast cancer (IBC) [1]. Over the years, the observed incidence of DCIS has increased significantly due to the popularization of mammography, currently accounting for approximately 20% of tumors detected through screening [2]. Without treatment, it is estimated that up to 40% of DCIS will transform into IBC [3]. However, the question for each individual case of tumor is whether complementary radiotherapy and hormone therapy are required in addition to surgical treatment. Therefore, it is essential to look for biomarkers that predict the probability of progression from DCIS to IBC. Neoductgenesis is a recently defined DCIS feature associated with a few histological determinants of DCIS aggressiveness, such as malignant-type calcifications, nuclear grade 3, negativity for hormone receptors, human epidermal growth factor receptor 2 (HER2) overexpression and higher proliferation index [4,5]. The recognition of neoductgenesis is based on three characteristics: concentration of ducts, lymphocytic infiltration (LI) and periductal fibrosis, all assessed on a 0–2 scale. However, prognostic significance and cut-off values are still under investigation [4,5,6].

Dendritic cells (DCs) are a diverse group of antigen-presenting cells. Located in tissues such as skin, gastrointestinal mucosa and lungs, DCs are in contact with the external environment. DCs are an extremely important link between innate immunity and adaptive immunity, playing a crucial role in both immune defense and the maintenance of immune tolerance. There are different DCs subtypes, namely conventional DCs, plasmacytoid DCs and monocyte-derived DCs [7]. Cluster of differentiation 1a (CD1a) is considered a universal marker of DCs, although it is mainly used to recognize immature cells. It is expressed on the surface of Langerhans cells and tumor-infiltrating DCs infiltrating the tumor [7,8]. Functionally, CD1a presents antigens derived from glycolipids and lipids and is involved in the activation of T cells [7]. Cluster of differentiation 123 (CD123) is a marker of plasmacytoid DCs, which are present in the peripheral blood and in inflammatory lymph nodes. The dendritic-cell-lysosome-associated membrane glycoprotein (DC-LAMP3 or CD208) is a marker of mature DCs that appears relatively late during their differentiation [8,9]. DC-LAMP^+^ DCs express multiple ligands to interact with receptors on T cells, possibly being the most active immune regulators of lymphocytes. A close relationship between T_reg_ cell markers (i.e., transforming growth factor β, TGF-β) and DC-LAMP mRNA levels was shown [10]. Dendritic-cell-specific intercellular-adhesion-molecule-3-grabbing non-integrin (DC-SIGN or CD209) is a C-type lectin receptor that is selectively expressed on DCs and is considered to be another marker of their maturity [11,12].

DCs are observed in healthy breast stroma, where they are closely associated with the epithelium of lobular acini. They are particularly observed in the terminal ductal lobular unit (TDLU), the most common origin site of IBC. Their increase is already visible in benign lesions, where a higher density of DCs is observed than in healthy breast stroma [13]. DCs play an extremely important role in initiating the immune response, and their presence is essential for the immune system to combat tumors. DC-based vaccines have been clinically successful in treating breast tumors, particularly in DCIS, where cancer cells and the immune system reach a state of equilibrium [14]. Martinez et al. suggested that the density of mature DC-LAMP^+^ DCs decreases as breast cancer (BC) progresses from DCIS to an invasive form [1]. In our previous study, we investigated the association of subpopulation density of DCs with molecular subtype, spatial location, hormone receptor status and clinical and histopathological prognostic factors in IBC. We showed the possible association of different DCs subpopulations expressing CD1a, cluster of differentiation 83 (CD83), CD123, DC-LAMP3 and DC-SIGN on their surface along with molecular subtypes of breast carcinoma, estrogen receptor (ER), progesterone receptor (PR) and progression-free survival [11].

We hypothesized that the different morphological features of DCIS may translate into different tumor immunogenicity and thus also to various types of antigenic stimulation of immune system cells. In such a situation, the assessment of DCs surface markers (i.e., indirectly, their particular subpopulations) may allow one to establish a relationship between the characteristics of infiltrating DCs and various morphological features of DCIS, including those of recognized prognostic importance. DCs seem to be particularly prominent candidates for such an assessment due to their role as professional antigen-presenting cells and important role in the development of anti-tumor immune response. The results of previous studies indicate that, in invasive cancers, various subtypes of DCs can be associated with patient prognosis [15], while analogous studies are lacking in the case of DCIS. Therefore, we decided to evaluate the subpopulations of DCs in preinvasive tumors.

The aim of our work is to assess the relationship between the densities of DCs populations expressing CD1a, CD123, DC-SIGN or DC-LAMP3 and the occurrence of several histopathological features that show prognostic significance in primary DCIS. In particular, we intended to investigate the relationship between the number of DCs in DCIS tumor tissue and the presence of neoductgenesis, which is a characteristic of DCIS associated with worse prognosis.

## 2. Results

### 2.1. Description of Study Group

The study group consisted of 92 female patients with breast DCIS, median age 56 years (range 31–85). The maximal tumor focus size ranged from 0.25 to 75.00 mm (median 13.00 mm). The clinicopathologic characteristics of the study group are summarized in Table 1.

**Table 1:** Clinicopathologic features of the study group. Quantitative data are given as median [min.-max. range] or mean ± standard deviation.

Characteristic			Missing
Age (years)	56 [31–85]		
Maximal tumor foci size (mm)	13.00 [0.25–75.00]		3
	**Architectural pattern (*N*, %)**		
Solid	69	75.0	
Cribriform	51	55.4	
Micropapillary	31	34.7	
Papillary	16	17.4	
Comedo	15	16.3	
Apocrine	7	7.6	
Clinging	1	1.1	
Spindle cell	1	1.1	
	**Highest nuclear grade (*N*, %)**		
G1	4	4.0	
G2	52	57.0	
G3	36	39.0	
ER (%)	53.94 ± 40.63		15
PR (%)	31.58 ± 36.85		15
	**Histological features (*N*, %)**		
Comedo necrosis	60	65.2	
Ductal spread	70	77.9	1
Lobular cancerization	48	52.7	1
Microinvasion	10	10.9	
Microcalcifications	72	78.3	
	**Neoductgenesis (*N*, %)**		
Neoductgenesis	26	28.0	
	**Other clinical features (*N*, %)**		
Family history of BC	13	17.3	17
The palpability of the lesions	18	25.4	21
Paget disease	10	10.9	

Abbreviations: BC—breast cancer, ER—estrogen receptor, G—nuclear grade, PR—progesterone receptor.

Regarding architectural pattern, the majority of the investigated DCIS cases showed mixed morphology (62 cases, 67.4%). Of the study group, nine (9.8%) displayed four, eighteen (19.6%) displayed three, thirty-five (38.0%) showed two architectural patterns and the remaining thirty (32.6%) showed a single morphological type. The most frequently recognized morphological patterns were solid (*N* = 69, 75.0%) and cribriform (*N* = 51, 55.4%).

Neoductgenesis was identified in 26 (28.0%) cases. Concentration of ducts was evaluated as 0 in 14 (15.2%), 1 in 67 (72.8%) and 2 in 11 (12.0%) cases. The intensity of lymphocytic infiltrate (LI) was 0 in 29 (31.5%), 1 in 47 (51.1%) and 2 in 16 (17.4%) cases. The distribution of the fibrosis score was 0 points in 45 (48.9%), 1 in 25 (27.2%) and 2 in 22 (23.9%) cases.

Family history of BC occurred in 13 (17.3%) patients. Paget disease was noted in 10 (10.9%) cases.

### 2.2. Relationships between DCs Densities and Histopathological Features of DCIS

**Figure 1:**
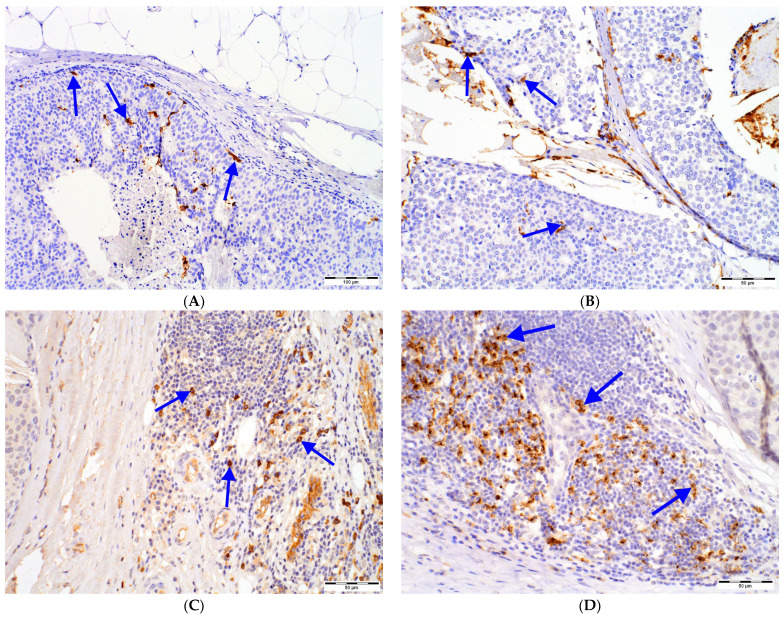

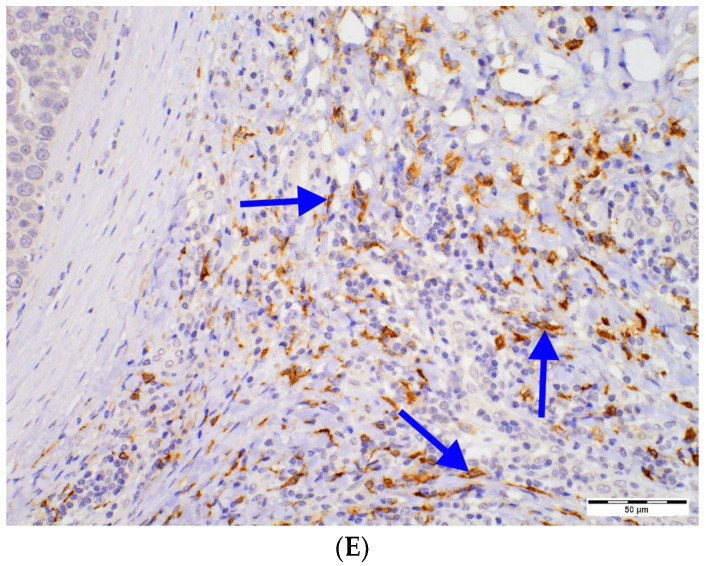
DCs in DCIS tissue samples: (**A**) intratumoral (magn. 100×) and (**B**) peritumoral (magn. 200×) CD1a^+^ DCs, (**C**) CD123^+^ cells (magn. 200×) located in immune infiltrate in the peritumoral area of DCIS (left), (**D**) DC-LAMP^+^ DCs located in immune infiltrate in the peritumoral area of DCIS (upper right; magn. 200×), (**E**) DC-SIGN^+^ DCs located in immune infiltrate in the peritumoral area of DCIS (upper left; magn. 200×); intensively stained DCs (blue arrows) need to be distinguished from nonspecific staining of other cells. Abbreviations: CD1a—cluster of differentiation 1a, CD123—cluster of differentiation 123, DCs—dendritic cells, DC-LAMP—dendritic-cell-lysosome-associated membrane glycoprotein, DC-SIGN—dendritic-cell-specific intercellular-adhesion-molecule-3-grabbing non-integrin, DCIS—ductal carcinoma in situ, magn.—magnification of microscope.

The investigated DCs populations were distributed primarily in the tumor surrounding stroma. However, for CD1a^+^ DCs, a prominent infiltrate was observed in the intratumoral area (Figure 1).

As the majority of the investigated DCIS show a mixed (≥2) architectural pattern, we investigated differences in DCs infiltrates between tumors, showing individual histological features versus the remaining cases (Table 2). Paget disease was associated with remarkably higher amounts of peritumoral CD1a^+^ DCs than in the case of its absence (p^BH^ < 0.001). DC-LAMP^+^ DCs were more abundant in comedo-type DCIS (p^BH^ < 0.001), while, in cribriform tumors, their number was lower (p^BH^ = 0.009) compared to the other cases (Table 2).

**Table 2:** Relationships between number of cells (*N*) of investigated DCs subpopulations and different histological features in DCIS. DCs numbers are expressed as median and interquartile range (Me [Q1–Q3]).

**CD1a^+^** **Peritumoral** **Cell Number [N]**	**Paget Disease**		
	**Absent**	**Present**	**p/p^BH^**
	4 [0–57]	218 [91–311]	<0.001/<0.001
**DC-LAMP^+^** **peritumoral cell number [N]**	**Cribriform architectural type**		
	**Absent**	**Present**	**p/pBH**
	123 [65–176]	30 [4–107]	0.002/0.009
	**Comedo architectural type**		
	**Absent**	**Present**	**p/pBH**
	61 [6–119]	177 [154–351]	<0.001/<0.001
	**Comedo necrosis**		
	**Absent**	**Present**	**p/pBH**
	28 [6–103]	87 [24–177]	0.007/0.02
	**Ductal spread**		
	**Absent**	**Present**	**p/pBH**
	7 [2–18]	107 [45–172]	<0.001/<0.001
	**Lobular cancerization**		
	**Absent**	**Present**	**p/pBH**
	37 [3–119]	107 [48–193]	0.002/0.009

The Mann–Whitney *U* test was performed. Results statistically significant after Benjamini–Hochberg corrections are presented; all outcomes of the analysis are shown in Supplementary Table S1. Abbreviations: CD1a—cluster of differentiation 1a, DC—dendritic cell, DCIS—ductal carcinoma in situ, DC-LAMP—dendritic-cell-lysosome-associated membrane glycoprotein, p/p^BH^—*p*-value and *p*-value after Benjamini–Hochberg correction (respectively).

Regarding other histopathological features of DCIS, the higher number of tumor-infiltrating DCs expressing DC-LAMP coexisted with the presence of ductal spread (p^BH^ < 0.001), lobular cancerization (p^BH^ = 0.009) and comedo necrosis (p^BH^ = 0.02) (Table 2).

No other relationship between DCIS architectural type or other histological features and DCs superficial markers was established. The complete results of analysis are shown in Supplementary Table S1.

**Figure 2:**
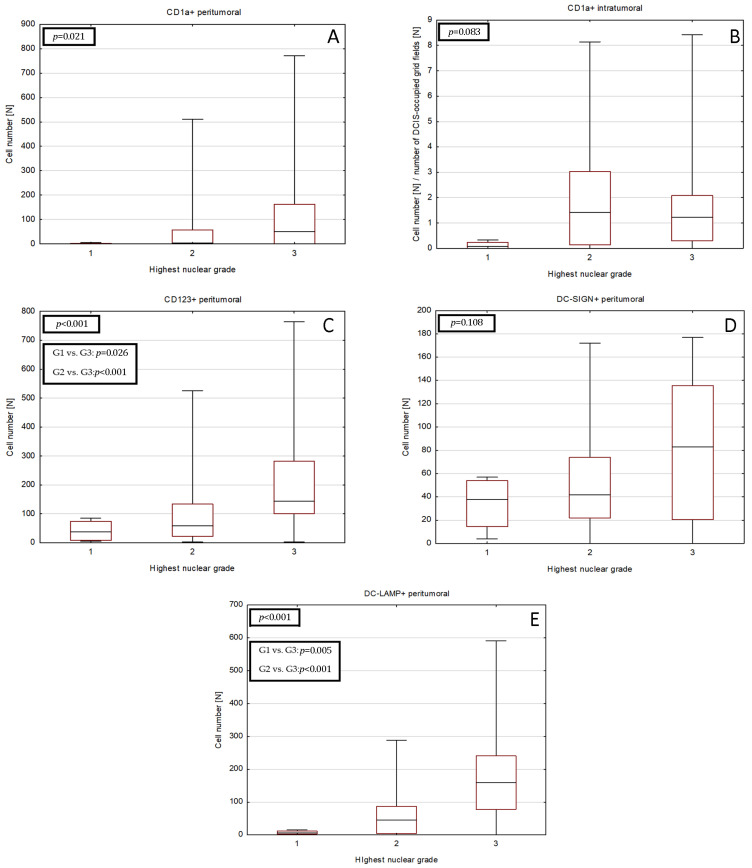
Differences in densities of DCs subset infiltrate in DCIS tumors of various nuclear grades. DCs subsets shown are as follows: (**A**) peritumoral CD1a^+^, (**B**) intratumoral CD1a^+^, (**C**) peritumoral CD123^+^, (**D**) peritumoral DC-SIGN^+^ and (**E**) peritumoral DC-LAMP^+^ DCs. The central point is median, box is interquartile range and whiskers are min–max range. Abbreviations: CD1a—cluster of differentiation 1a, CD123—cluster of differentiation 123, DCs—dendritic cells, DC-LAMP—dendritic-cell-lysosome-associated membrane glycoprotein, DC-SIGN—dendritic-cell-specific intercellular-adhesion-molecule-3-grabbing non-integrin, DC—dendritic cell, DCIS—ductal carcinoma in situ.

Combination of two nuclear grades in a single patient was frequently observed within our study group. Therefore, we investigated the relationships between the densities of DCs and the highest (worst) grade observed in each case (Figure 2, Table S2). Peritumoral CD1a^+^, CD123^+^ and DC-LAMP^+^ cell numbers were associated with higher grade, although significant intergroup relationships were observed only for the CD123^+^ and DC-LAMP^+^.

### 2.3. Relationships between DCs Densities and Neoductgenesis

In DCIS, neoductgenesis is characterized by the presence and intensity of three features: concentration of ducts, LI, and fibrosis. Differences between DCs density and each neoductgenesis hallmark were investigated with Kruskal–Wallis analysis of variance (ANOVA) test with post hoc multiple comparison of average ranks (Table 3). The density of peritumoral CD1a^+^ DCs was lower in cases with higher concentration of ducts; however, the significance was borderline (p^BH^ = 0.048) and post hoc test revealed the difference between tumors that scored 0 vs. 1 but not between those that scored 0 vs. 2. All populations of DCs, except for DC-SIGN^+^, were associated with higher scores for LI. Post hoc test showed a trend between LI score and densities of both CD123^+^ and DC-LAMP^+^. The density of CD123^+^ and DC-LAMP^+^ DCs was also increased in tumors with features of fibrosis.

Tumors in which recognition of neoductgenesis was established were infiltrated by higher amounts of CD123^+^ and DC-LAMP^+^ DCs.

### 2.4. Correlations between DCs Densities and Tumor Size or HR Expression

**Figure 3:**
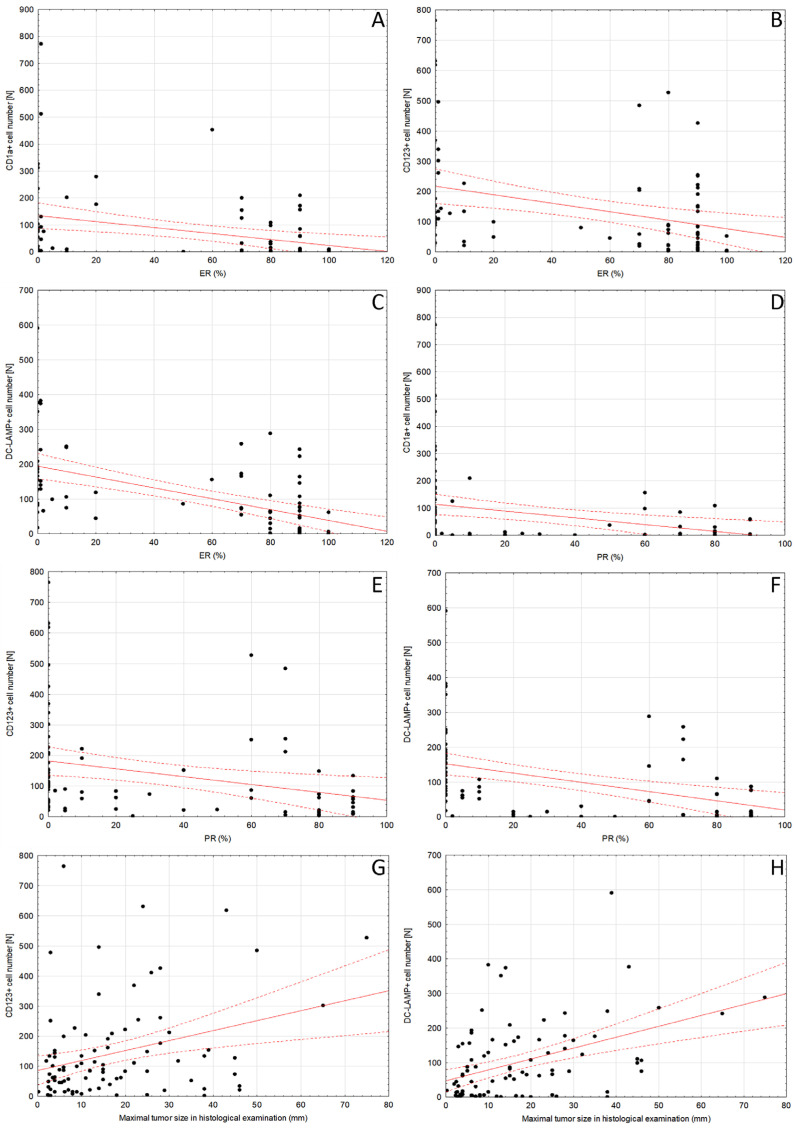
Scatter plot diagrams of significant relationships between (**A**) peritumoral CD1a^+^ DCs number and ER expression, (**B**) CD123^+^ DCs number and ER expression, (**C**) DC-LAMP^+^ DCs number and ER expression, (**D**) peritumoral CD1a^+^ DCs number and PR expression, (**E**) CD123^+^ DCs number and PR expression, (**F**) DC-LAMP^+^ DCs number and PR expression, (**G**) CD123^+^ DCs number and maximal tumor size in histological examination and (**H**) DC-LAMP^+^ DCs number and maximal tumor size in histological examination. Trend line is indicated in red, and the 95% confidence interval is marked by the dotted lines. Spearman R coefficients and *p*-values are shown in Table 4. Abbreviations: CD1a—cluster of differentiation 1a, CD123—cluster of differentiation 123, DC—dendritic cell, DC-LAMP—dendritic-cell-lysosome-associated membrane glycoprotein, ER—estrogen receptor, PR—progesterone receptor, DC—dendritic cell.

The number of peritumoral CD1a^+^, CD123^+^ and DC-LAMP^+^ DCs showed moderate negative correlation with expression of both ER and PR. Furthermore, there was a weak to moderate correlation between the maximum tumor size in histological examination and the number of CD123^+^ and DC-LAMP^+^ DCs (Table 4, Figure 3). No significant relationship with tumor size in ultrasound imaging or mammography was found. The complete correlation matrix is presented in Supplementary Table S3.

### 2.5. Univariate Logistic Regression Analysis of Relationship between DCs Subpopulations and DCIS Histological Features

A series of simple predictive models of univariate logistic regression was constructed to further investigate the relationships indicated by the Mann–Whitney *U* test, namely between the amount of peripheral CD1a^+^ DCs and Paget disease as well as between DC-LAMP^+^ DCs and architectural type (cribriform, comedo) and histological characteristics (comedo necrosis, ductal spread, lobular cancerization) (Table 5). Due to the data distribution, the arbitrary cut-off point for CD1a^+^ DCs was set to one-hundred stained cells in five HPFs, while, for the DC-LAMP^+^ DCs, the increment of ten cells in five HPFs was chosen to be the predictor.

**Table 3:** Relationships between densities of investigated DCs subpopulations and determinants of neoductgenesis in DCIS. The intensity of the concentration of the ducts, lymphocytic infiltrate and fibrosis was scored 0–2 according to Zhou et al. [4,5]. “Neoductgenesis” was determined if the tumor was scored 4 points or more. The Benjamini–Hochberg correction was applied.

Neoductgenesis Feature Score	CD1a^+^ Intratumoral Cell Number ^$^ [N]	p/p^BH^	CD1a^+^ Peritumoral Cell Number [N]	p/p^BH^	CD123^+^ Peritumoral Cell Number [N]	p/p^BH^	DC-SIGN^+^ Peritumoral Cell Number [N]	p/p^BH^	DC-LAMP^+^ Peritumoral Cell Number [N]	p/p^BH^
**Concentration of ducts (score)**										
0	1.7 [0.1–5.0]	0.672/-	116 [3–235] *	0.032/ 0.048	115.5 [45–203]	0.064/-	38 [27–105]	0.909/-	64 [13–154]	0.305/-
1	1.2 [0.1–2.3]		4 [0–81] *		97 [40–191]		46 [20–94]		76 [15–176]	
2	1.6 [0.1–1.9]		5 [0–57]		51 [10–84]		46 [22–96]		9 [5–87]	
**Lymphocytic infiltration (score)**										
0	0.1 [0–1.6] *,#	0.002/ 0.005	0 [0–4] *,#	<0.001/ <0.001	51 [15–85] *,#	<0.001/ <0.001	45 [25–58]	0.132/-	7 [2–18] *,#	<0.001/ <0.001
1	1.7 [0.4–3.5] *		14 [2–91] *		87 [40–199] *,#,†		42 [13–104]		87 [45–167] *,#,†	
2	1.7 [1.1–2.5] #		86 [18–164] #		184 [139–455] #,†		94 [16–136]		169 [128–316] #,†	
**Fibrosis (score)**										
0	1.2 [0–2.7]	0.176/-	3 [0–57]	0.312/-	59 [15–134] *	0.012/ 0.018	40 [19–75]	0.575/-	14 [3–76] *,#	<0.001/ <0.001
1	2.2 [1–3]		21 [2–81]		102 [61–176]		50 [31–94]		87 [37–164] *	
2	1.4 [0–1.7]		50 [0–155]		158 [52–302] *		53 [9–130]		174 [86–248] #	
	**CD1a+** **intratumoral** **cell number $ [N]**	***p*-value**	**CD1a+** **peritumoral** **cell number [N]**	***p*-value**	**CD123+** **peritumoral** **cell number [N]**	***p*-value**	**DC-SIGN+** **peritumoral** **cell number [N]**	***p*-value**	**DC-LAMP+** **peritumoral** **cell number [N]**	***p*-value**
**Neoductgenesis**										
present	1.6 [0.2–1.9]	0.857	50 [0–102]	0.185	158 [80–339]	0.002	53 [13–124]	0.728	174 [87–248]	<0.001
absent	1.3 [0.1–2.7]		4 [0–76]		69 [23–134]		45 [22–76]		41 [4–107]	

The Mann–Whitney *U* test (for comparisons between 2 variables) or the ANOVA Kruskal–Wallis test (for comparisons between 3 variables) were performed. DCs numbers are expressed as median and interquartile range (Me [Q1–Q3]). Symbol (^$^) indicates that the number of CD1a^+^ DCs was averaged per number of 3 × 3 grid fields (field size: 1023 × 767.5 µm/1637 × 1228 pixels) occupied by DCIS foci. Symbols (*, ^#^, ^†^) indicate significant differences between given groups found via post hoc multiple comparison of average ranks. Abbreviations: CD1a—cluster of differentiation 1a, CD123—cluster of differentiation 123, DC—dendritic cell, DCIS—ductal carcinoma in situ, DC-LAMP—dendritic-cell-lysosome-associated membrane glycoprotein, DC-SIGN—dendritic-cell-specific intercellular-adhesion-molecule-3-grabbing non-integrin, p/p^BH^—*p*-value and *p*-value after Benjamini–Hochberg correction (respectively).

**Table 4:** Correlations between number of cells (*N*) of investigated DCs subpopulations and nuclear receptors expression or maximal tumor size of DCIS.

	Estrogen Receptor Expression (%)	
	Spearman R	p/p^BH^
**CD1a^+^ peritumoral**	−0.38	<0.001/0.002
**CD123^+^ peritumoral**	−0.43	<0.001/<0.001
**DC-LAMP^+^** **peritumoral**	−0.62	<0.001/<0.001
	**Progesterone receptor expression (%)**	
	**Spearman R**	**p/p^BH^**
**CD1a^+^ peritumoral**	−0.42	<0.001/<0.001
**CD123^+^ peritumoral**	−0.48	<0.001/0.001
**DC-LAMP^+^** **peritumoral**	−0.59	<0.001/<0.001
	**Maximal tumor size in histological examination (mm)**	
	**Spearman R**	**p/p^BH^**
**CD123^+^ peritumoral**	0.29	0.007/0.01
**DC-LAMP^+^** **peritumoral**	0.42	<0.001/<0.001

Nonparametric Spearman correlation coefficient was calculated. Results statistically significant after Benjamini–Hochberg correction are presented; all outcomes of the analysis are shown in Supplementary Table S3. Abbreviations: CD1a—cluster of differentiation 1a, DC—dendritic cell, DCIS—ductal carcinoma in situ, DC-LAMP—dendritic-cell-lysosome-associated membrane glycoprotein, p/p^BH^—*p*-value and *p*-value after Benjamini–Hochberg correction (respectively).

**Table 5:** Univariate logistic regression for relationships between CD1a^+^ or DC-LAMP^+^ cell number and histological features selected based on the results shown in Table 2.

**Number of Peripheral CD1a^+^ Cells >100**	**Paget Disease**	
	**OR (95%CI)**	**p/p^BH^**
	13.22 (2.99–58.39)	<0.001/<0.001
**per 10 DC-LAMP^+^ cells increase**	**Cribriform architectural type**	
	**OR (95%CI)**	**p/p^BH^**
	0.95 (0.91–0.99)	0.017/0.017
	**Comedo architectural type**	
	**OR (95%CI)**	**p/p^BH^**
	1.13 (1.06–1.20)	<0.001/<0.001
	**Comedo necrosis**	
	**OR (95%CI)**	**p/p^BH^**
	1.08 (1.02–1.14)	0.010/0.012
	**Ductal spread**	
	**OR (95%CI)**	**p/p^BH^**
	1.21 (1.08–1.36)	0.001/0.003
	**Lobular cancerization**	
	**OR (95%CI)**	**p/p^BH^**
	1.08 (1.03–1.14)	0.003/0.005

The Benjamini–Hochberg correction was applied. Due to the data distribution, the arbitrary cut-off point for CD1a^+^ was set at 100 stained cells in 5 HPFs, while, for the DC-LAMP^+^, the increment of 10 cells in 5 HPFs was chosen to be the predictor. Abbreviations: CD1a—cluster of differentiation 1a, DC—dendritic cell, DCIS—ductal carcinoma in situ, DC-LAMP—dendritic-cell-lysosome-associated membrane glycoprotein, HPF—high-power field, OR (95%CI)—odds ratio (95% confidence interval), p/p^BH^—*p*-value and *p*-value after Benjamini–Hochberg correction (respectively).

Each of the investigated features was shown to be properly predicted by the models. If more than 100 CD1a^+^ DCs were counted, the odds of associated Paget disease were substantially higher (odds ratio: 13.22, 95% confidence interval: 2.99–58.39, p^BH^ < 0.001). Concerning the DC-LAMP^+^ DCs subpopulation, for each additional 10 cells, the odds of recognizing comedo DCIS, comedo necrosis, ductal spread and lobular cancerization were higher by 10–20%. Oppositely, the odds of finding cribriform DCIS were lower by 5% per each 10 DC-LAMP^+^ DCs identified.

The goodness-of-fit Hosmer–Lemeshow test was conducted, with *p* > 0.9 in each model.

## 3. Discussion

The components of the tumor microenvironment can contribute to cancer progression and dissemination of tumor cells [16,17,18,19]. It was shown that there is an increase in the number of multiple immune cells of the microenvironment from normal breast tissue to DCIS, suggesting that an active immune response occurs early during BC progression [13,20].

According to our observations, DC-LAMP^+^ DCs are associated with recognized histological factors of a worse prognosis in DCIS, such as central necrosis, high nuclear grade, certain architectural pattern, larger tumor size or lower expression of estrogen and progesterone receptors [21]. This is consistent with the study of Martinet et al., who showed that the number of mature DC-LAMP^+^ DCs in the tumor stroma drops with the transition from in situ to IBC, with a parallel decrease in the number of high endothelial venules [22]. A wide range of research investigated the role of DCs in IBC and provided evidence that DCs’ functions can be altered and converted from antitumor to immunoregulatory and tumor-supportive in cancer [16,19,23,24]. However, properly activated DCs are considered to exert a suppressive effect on BC. Zhong et al. conducted cell type enrichment analysis, which suggested that the DCs score is a predictor of long-term survival in BC and associated with characteristics such as molecular type, clinical stage and Ki-67 expression [25]. On the contrary, high abundance (above the median) of resting DCs is associated with worse prognosis [26]. Several molecules expressed or secreted by BC cells, such as polyamines [23], growth factors [27,28], interleukins and cytokines [28,29], were found to affect DCs properties.

To the best of our knowledge, research studies investigating associations between DCIS architectural pattern and DCs infiltration are scarce. This could be due to the observation that DCIS frequently displays mixed morphology [30], which impedes such comparisons. Perez et al. [30] found that factors associated with an increased risk of local recurrence, such as high-grade necrosis and comedo necrosis, are more prevalent in solid and are infrequently found in cribriform subtypes. We noted that the features of comedo-type DCIS are related to higher densities of DC-LAMP^+^ DCs. Opposingly, cribriform DCIS is characterized by lower numbers of mature DC-LAMP^+^ DCs. This suggests that DCIS morphological variants have differences in the infiltrate of the respective DCs populations and that maturity of the DCs themselves is not obligatorily associated with a more favorable DCIS type. In IBC, it was suggested that the paucity of mature DCs is due to failure in their recruitment, migration and maturation [31]. The latter process can be arrested by regulatory T cells, and, subsequently, low numbers of mature DCs may result from failed antigen presentation [7]. Moreover, the correlation between regulatory T cells and plasmacytoid DCs suggests a relationship between plasmacytoid DCs and immunosuppression [8].

In this study, we observed that the populations of peritumoral CD1a^+^, plasmacytoid CD123^+^ and DC-LAMP^+^ DCs are more numerous in G3 than in lower-grade DCIS. Previously, we showed that there is more infiltration of CD1a^+^ and plasmacytoid CD123^+^ DCs into high-grade IBC than into well-differentiated tumors [11]. Lopez et al. observed high densities of these and DC-LAMP^+^ cell populations in high-grade triple-negative BC [32]. In contrast, other authors suggested that there are associations between more mature DCs and low-grade IBC [27] or no relationship between CD1a^+^ DCs and IBC grade [27,31]. Therefore, it appears that the distribution of DCs subsets in tumors of different grades remains similar during the transition from DCIS to IBC.

As previously mentioned, comedo necrosis is an adverse prognostic factor in DCIS [30,33]. However, its predictive value and its features such as ductal spread are still under debate in DCIS [34,35]. We noted that comedo necrosis and lobular cancerization are related to a higher peritumoral infiltration of mature DCs. The higher numbers of CD1a^+^ DCs are also associated with Paget disease. It was observed by Brunhuber that mature and CD1a^+^ DCs are present close to the tumor area and may interact with Paget cells [36]. These results highlight that DCs are associated with several arguably prognostic factors in DCIS.

Tumor size is one of the key risk factors in DCIS [1,33]. In IBC, the higher numbers of CD1a^+^ and mature CD83^+^ DCs are associated with smaller tumor size [27,29], higher overall survival and a lower risk of relapse in triple-negative BC after neoadjuvant chemotherapy without pathological complete response [37]. Surprisingly, we observed that mature DC-LAMP^+^ DCs slightly increase with tumor diameter in DCIS. This would suggest a different role of DCs in the growth of invasive or in situ disease than in invasive tumors.

Negativity for ER and PR is considered a factor of progression of DCIS to IBC [14]. Kovats et al. suggested that ER signaling can influence DCs function [38]. DCs express ER on their surface. The estrogenic stimulation of immune system cells may lead to their proliferation and the production of cytokines; however, it also stimulates the production of growth factors [13,38]. Several studies show that higher numbers of immature CD1a^+^ DCs are associated with hormone receptor negativity in IBC [27], which is in line with our study in DCIS.

DCIS is a preinvasive form of BC; however, its treatment is based on the surgical removal of the tumor accompanied by radiation therapy or hormone therapy. It is debatable whether all these changes require such an advanced treatment after the diagnosis; some may only require observation [5]. The search for possible tumor characteristics is under way to divide DCIS into those requiring rapid intervention due to the high risk of invasiveness and those that can be monitored.

The theory of neoductgenesis was originally proposed by the radiologist Laszlo Tabar. It describes the features of DCIS associated with a worse prognosis [39]. Neoductgenesis leads to the formation of tumors that contain new pathological ducts. Zhou et al. established a pathological classification that describes the characteristics of tumors with neoductgenesis, which requires evaluation of duct concentration, LI and periductal fibrosis (each of these parameters on a scale of 0–2) [4]. Both Zhou et al. and our team found an appropriate cut-off point to be a score of 4–6 [5,6]. Additionally, they showed that neoductgenesis characteristics are associated with determinants of a poor prognosis compared with other cases [4]. Tumors with neoductgenesis frequently demonstrate low expression of ER and PR as well as overexpression of HER2 [4], indicating that they may be precursors of non-luminal cancers. Our recent study suggested that neoductgenesis in DCIS is related to multiple morphological characteristics typical for tumors of aggressive biology [6].

In this study, we showed a significant relationship between neoductgenesis and both CD123^+^ and DC-LAMP^+^ DCs. We were the first to strictly evaluate the relationship between neoductgenesis in DCIS and the distribution of DCs subpopulations.

A higher density of CD123^+^ and DC-LAMP^+^ cells was also associated with a denser LI and a higher intensity of periductal fibrosis. Plasmacytoid DCs have been detected in disease processes characterized by severe fibrosis, such as idiopathic pulmonary fibrosis and systemic sclerosis [40]. In animal models, the presence of these cells in the lungs is correlated with the severity of systemic sclerosis [41]. Therefore, we hypothesize that peripheral fibrosis observed in DCIS with neoductgenesis characteristics is aggravated by plasmacytoid DCs. The more aggressive tumors would be infiltrated by a larger amount of plasmacytoid DCs, and then the intensity of observed fibrosis would be higher.

According to the observations of Wang et al., a higher expression of DC-LAMP was visible in liver biopsies of patients suffering from chronic hepatitis B virus (HBV) infection. Upregulation of DC-LAMP was associated with T cell activation and adaptive immune regulation. There was a close relationship between the expression of the DC-LAMP TGF-β gene [10], which is an important factor leading to the intensification of fibrosis processes [42].

It should be noted that DCs are not only investigated in the scope of diagnosis but can be stimulated, to enhance their natural function, through vaccines (e.g., prepared as exosomes containing immunogenic cell death inducers) [43]. Alternatively, DCs can be stimulated with tumor antigens ex vivo and then administered to the subject with the intention of aiding natural immunity [44]. DC-based vaccines are considered safe preparations, and their use in cancer immunotherapy is the subject of ongoing research [45]. Multiple studies have shown that DC-based treatments might have a potential effect on BC. The clinical study of a DCs vaccine in BC has demonstrated considerable anti-tumor effects, and some DCs vaccines undergo assessment in clinical trials [46,47].

## 4. Material and Methods

### 4.1. Patient Selection

The material consisted of 92 routinely processed, formalin-fixed, paraffin-embedded primary DCIS tumor archival samples diagnosed between 2008 to 2021 that were retrospectively evaluated. Slides were reevaluated by a histopathologist experienced in breast diagnosis. The diagnosis of DCIS in the surgically excised specimen and female gender was the basis for inclusion in the study. The exclusion criteria for the study were the coexistence of invasive cancer larger than microinvasive carcinoma and the prior diagnosis of invasive cancer in the same breast, as well as neoadjuvant treatment.

### 4.2. Immunohistochemical Techniques

Immunohistochemistry for CD1a, CD123, DC-LAMP, DC-SIGN, ER and PR was performed according to the protocol routinely used in our laboratory (Table 6). Tonsil tissue served as both a negative and positive control for immunohistochemistry. Positive expression of ER and PR was set at ≥1% of tumor cells showing positive nuclear immunostaining.

**Table 6:** Antibodies used in the study.

Antibody	Clone	Dilution	Antigen Retrieval	Incubation Time	Manufacturer	Detection System
CD1a	MTB1	8:100	Citrate	60 min	Novocastra	Immunologic
CD123	BR4MS	1:100	EDTA	30 min	Novocastra	Immunologic
DC-LAMP	polyclonal	1:50	EDTA	30 min	Novus	Immunologic
DC-SIGN	5D7	1:50	EDTA	60 min	Abcam	Immunologic
ER	SP1	RTU	Citrate	30 min	Roche	Ultra Vision Dab Detection Kit
PR	1E2	RTU	Citrate	60 min	Roche	Ultra Vision Dab Detection Kit

Abbreviations: CD1a—cluster of differentiation 1a, CD123—cluster of differentiation 123, DC-LAMP—dendritic-cell-lysosome-associated membrane glycoprotein, DC-SIGN—dendritic-cell-specific intercellular-adhesion-molecule-3-grabbing non-integrin, EDTA—ethylenediaminetetraacetic acid, ER—estrogen receptor, min—minutes, PR—progesterone receptor.

### 4.3. Histologic Evaluation, DCs Scoring and Analysis

Nuclear grade, architectural pattern and other histological features of DCIS were evaluated according to College of American Pathologists protocols. Nuclear grade was determined using 6 morphologic features, such as pleomorphism, size of nuclei and nucleoli, chromatin distribution, mitoses and cell orientation [48]. The architectural pattern was based on morphology: cribriform DCIS was defined based on sieve-like proliferation of neoplastic cells, solid DCIS was defined based on solid proliferation of neoplastic cells, micropapillary pattern was characterized by bulbous epithelial projections into the duct lumen without fibrovascular cores, papillary DCIS showed intraductal branching projections with fibrovascular cores, comedo-type was characterized by central necrosis and high-grade nuclei, apocrine DCIS was composed of cells with voluminous pink cytoplasm and visible granularity, clinging pattern was characterized by a single layer of highly atypical cells and spindle cell DCIS was composed of spindle cells. Comedo necrosis was defined as expansive dirty necrosis with visible ghost cells in the central part of the affected ducts. Microinvasion was defined as invasive carcinoma not greater than 1 mm in any dimension [48]. The features of neoductgenesis were evaluated on the basis of the classification proposed by Zhou et al. [4], which scores three parameters: (i) concentration of ducts, (ii) lymphocytic infiltrate and (iii) fibrosis. Scoring 4 points or more was considered as the criterion for neoductgenesis [5,6].

The immunostained slides were initially scanned on Olympus BX53 optical microscope (Olympus Corporation, Tokyo, Japan) at low magnification (100×), and the areas with the highest number of cells positive for CD1a, CD123, DC-SIGN and DC-LAMP were chosen. Morphological features of DCs subsets were included: CD1a^+^, DC-LAMP3^+^ and DC-SIGN DCs showed dendritic appearance, while CD123^+^ DCs presented as round cells without protrusions (Figure 1). Moreover, only cells with strong cytoplasmic staining and visible nuclei were counted to avoid overestimating due to unspecific staining. Then, digital microphotographs of 5 high-power fields (HPFs; 400×) in non-overlapping areas were taken using Olympus SC180 camera (Olympus Corporation, Tokyo, Japan). Positively stained DCs populations were counted in microphotographs with the use of Olympus CellSens Standard 2.3 software (Olympus Corporation, Tokyo, Japan) and its Object Counting tool. Cell counts obtained in 5 microphotographs were added. The CD1a^+^, CD123^+^, DC-SIGN^+^ and DC-LAMP^+^ cells located in tumor surrounding stroma no further than 1 HPF from the tumor edge were counted and regarded as peritumoral. The number of CD1a^+^ DCs was also evaluated within DCIS foci and regarded as intratumoral. To eliminate the impact of the size of the DCIS foci on number of intratumoral CD1a^+^ DCs, the Grid tool was used, and the number of CD1a^+^ DCs was averaged per number of 3 × 3 grid fields (field size: 1023 × 767.5 µm/1637 × 1228 pixels) occupied by DCIS foci.

### 4.4. Statistical Analysis

Data for categorical variables are presented as frequencies (*N*) and proportions (%), while, for interval variables, they are presented as mean ± standard deviation or median with range (min–max. or interquartile) according to data distribution type, as indicated by the Shapiro–Wilk test for normality with additional visual assessment of histograms. If any data were missing, the given case was not proceeded in the analysis for the given variable.

The lack of normality across the groups in conducted comparisons imposed the application of nonparametric tests. Thus, *U* Mann–Whitney test was used to investigate difference between two groups and Kruskal–Wallis ANOVA test was performed for three groups. Multiple comparison of average ranks was then applied as post hoc test to identify intergroup differences. Spearman’s correlation coefficient was used to describe relationships between the data from the interval. Univariate logistic regression models were proposed on the identified differences between the groups.

The significance threshold in all tests was α = 0.05. The Benjamini–Hochberg correction for multiple comparisons was used where applicable with the assumption of FDR = 0.05. Statistical analysis was performed with Statistica 13.3 software (Statsoft Inc., Tulsa, OK, USA).

## 5. Conclusions

DCs play an important role in establishing tumor-killing or tumor-promoting immune responses and are a promising target in novel BC immunotherapy. However, the data regarding a relationship between DCs infiltration and preinvasive BC are modest. Our results highlight that DCs densities show an association with DCIS growth and that their subsets are related to several unfavorable factors in preinvasive breast tumors as well as their architectural pattern and ER or PR expression. Moreover, DCs appear to be associated with another novel, presumably adverse, feature of DCIS—neoductgenesis—and its determinants: presence of fibrosis, concentration of ducts and LI. One should bear in mind the limitations of the study, which include a relatively small and heterogeneous group of patients. Further studies are needed to fully elucidate the cause and effect link between densities of DCs subpopulations, their maturity status and breast tumor progression from DCIS to IBC.

## Data Availability

The data presented in this study are available on request from the corresponding author. The data are not publicly available due to privacy restrictions.

## Supplementary Materials

The following supporting information can be downloaded at: https://www.mdpi.com/article/10.3390/ijms24129918/s1.

## Abbreviations

ANOVA	analysis of variance
BC	breast cancer
CD1a	cluster of differentiation 1a
CD83	cluster of differentiation 83
CD123	cluster of differentiation 123
DCIS	ductal carcinoma in situ
DCs	dendritic cells
DC-LAMP	dendritic-cell-lysosome-associated membrane glycoprotein
DC-SIGN	dendritic-cell-specific intercellular-adhesion-molecule-3-grabbing non-integrin
DNA	deoxyribonucleic acid
ER	estrogen receptor
HBV	hepatitis B virus
HER2	human epidermal growth factor receptor 2
HPFs	high-power fields
IBC	invasive breast cancer
LI	lymphocytic infiltration
PR	progesterone receptor
TDLU	terminal ductal lobular unit
TGF-β	transforming growth factor β

## References

[1] Martínez-Pérez C., Turnbull A.K., Ekatah G.E., Arthur L.M., Sims A.H., Thomas J.S., Dixon J.M. (2017). Current treatment trends and the need for better predictive tools in the management of ductal carcinoma in situ of the breast. Cancer Treat Rev..

[2] Dettogni R.S., Stur E., Laus A.C., da Costa Vieira R.A., Marques M.M.C., Santana I.V.V., Pulido J.Z., Ribeiro L.F., de Jesus Parmanhani N., Agostini L.P. (2020). Potential biomarkers of ductal carcinoma in situ progression. BMC Cancer.

[3] McCormick B., Winter K., Hudis C., Kuerer H.M., Rakovitch E., Smith B.L., Sneige N., Moughan J., Shah A., Germain I. (2015). RTOG 9804: A prospective randomized trial for goodrisk ductal carcinoma in situ comparing radiotherapy with observation. J. Clin. Oncol..

[4] Zhou W., Sollie T., Tot T., Pinder S.E., Amini R.-M., Blomqvist C., Fjällskog M.-L., Christensson G., Abdsaleh S., Wärnberg F. (2014). Breast Cancer with Neoductgenesis: Histopathological Criteria and Its Correlation with Mammographic and Tumour Features. Int. J. Breast Cancer..

[5] Zhou W., Sollie T., Tot T., Blomqvist C., Abdsaleh S., Liljegren G., Wärnberg F. (2017). Ductal Breast Carcinoma in Situ: Mammographic Features and Its Relation to Prognosis and Tumour Biology in a Population Based Cohort. Int. J. Breast Cancer.

[6] Łazarczyk A., Streb J., Hałubiec P., Streb-Smoleń A., Jach R., Hodorowicz-Zaniewska D., Łuczyńska E., Szpor J. (2023). Ne-oductgenesis in Ductal Carcinoma In Situ Coexists with Morphological Abnormalities Characteristic for More Aggressive Tumor Biology. Diagnostics.

[7] Liu J., Zhang X., Cheng Y., Cao X. (2021). Dendritic cell migration in inflammation and immunity. Cell Mol. Immunol..

[8] Mansfield A.S., Heikkila P., von Smitten K., Vakkila J., Leidenius M. (2011). Metastasis to sentinel lymph nodes in breast cancer is associated with maturation arrest of dendritic cells and poor co-localization of dendritic cells and CD8+ T cells. Virchows Arch..

[9] Szpor J., Streb J., Glajcar A., Sadowski P., Streb-Smoleń A., Jach R., Hodorowicz-Zaniewska D. (2022). Presence of Dendritic Cell Subsets in Sentinel Nodes of Breast Cancer Patients Is Related to Nodal Burden. Int. J. Mol. Sci..

[10] Wang Z., Wang X., Jin R., Liu F., Rao H., Wei L., Chen H., Feng B. (2023). LAMP3 expression in the liver is involved in T cell activation and adaptive immune regulation in hepatitis B virus infection. Front. Immunol..

[11] Szpor J., Streb J., Glajcar A., Frączek P., Winiarska A., Tyrak K.E., Basta P., Okoń K., Jach R., Hodorowicz-Zaniewska D. (2021). Dendritic cells are associated with prognosis and survival in breast cancer. Diagnostics.

[12] Merlotti A., Dantas E., Remes Lenicov F., Ceballos A., Jancic C., Varese A., Rubione J., Stover S., Geffner J., Sabatté J. (2015). Fucosylated clusterin in semen promotes the uptake of stress-damaged proteins by dendritic cells via DC-SIGN. Hum. Reprod..

[13] Goff S.L., Danforth D.N. (2021). The Role of Immune Cells in Breast Tissue and Immunotherapy for the Treatment of Breast Cancer. Clin. Breast Cancer..

[14] Zachariah N.N., Basu A., Gautam N., Ramamoorthi G., Kodumudi K.N., Kumer N.B., Loftus L., Czerniecki B.J. (2021). Intercepting Premalignant, Preinvasive Breast Lesions Through Vaccination. Front. Immunol..

[15] Stovgaard E.S., Nielsen D., Hogdall E., Balslev E. (2018). Triple negative breast cancer—Prognostic role of immune-related factors: A systematic review. Acta Oncol..

[16] Bates J.P., Derakhshandeh R., Jones L., Webb T.J. (2018). Mechanisms of immune evasion in breast cancer. BMC Cancer.

[17] Anstine L.J., Keri R. (2019). A new view of the mammary epithelial hierarchy and its implications for breast cancer initiation and metastasis. J. Cancer Metastasis Treat..

[18] Gatti-Mays M.E., Balko J.M., Gameiro S.R., Bear H.D., Prabhakaran S., Fukui J., Disis M.L., Nanda R., Gulley J.L., Kalinsky J. (2019). If we build it they will come: Targeting the immune response to breast cancer. npj Breast Cancer.

[19] Shihab I., Khalil B.A., Elemam N.M., Hachim I.Y., Hachim M.Y., Hamoudi R.A., Maghazachi A.A. (2020). Understanding the role of innate immune cells and identifying genes in breast cancer microenvironment. Cancers.

[20] Nelson A.C., Machado H.L., Schwertfeger K.L. (2018). Breaking through to the Other Side: Microenvironment Contributions to DCIS Initiation and Progression. J. Mammary Gland Biol. Neoplasia.

[21] WHO Classification of Tumours Editorial Board (2019). WHO Classification of Tumours.

[22] Martinet L., Filleron T., le Guellec S., Rochaix P., Garrido I., Girard J.-P. (2013). High Endothelial Venule Blood Vessels for Tumor-Infiltrating Lymphocytes Are Associated with Lymphotoxin β–Producing Dendritic Cells in Human Breast Cancer. J. Immunol..

[23] Gervais A., Levêque J., Bouet-Toussaint F., Burtin F., Lesimple T., Sulpice L., Patard J.-J., Genetet N., Catros-Quemener V. (2005). Dendritic cells are defective in breast cancer patients: A potential role for polyamine in this immunodeficiency. Breast Cancer Res..

[24] da Cunha A., Michelin M.A., Murta E.F.C. (2014). Pattern response of dendritic cells in the tumor microenvironment and breast cancer. World J. Clin. Oncol..

[25] Zhong S., Jia Z., Zhang H., Gong Z., Feng J., Xu H. (2021). Identification and validation of tumor microenvironment-related prognostic biomarkers in breast cancer. Transl. Cancer Res. TCR.

[26] Dai Q., Wu W., Amei A., Yan X., Lu L., Wang Z. (2021). Regulation and characterization of tumor-infiltrating immune cells in breast cancer. Int. Immunopharmacol..

[27] El Deeb N.M.F., Mehanna R.A. (2013). Assessment of Maturation Status of Tumor-Infiltrating Dendritic Cells in Invasive Ductal Carcinoma of the Breast: Relation with Vascular Endothelial Growth Factor Expression. Turk Patoloji Derg. Turk. J. Pathol..

[28] Sisirak V., Vey N., Goutagny N., Renaudineau S., Malrfroy M., Thys S., Treilleux I., Intidhar Labidi-Galy S., Bachelot T., Dezutter-Dambuyant C. (2013). Breast cancer-derived transforming growth factor-β and tumor necrosis factor-α compromise interferon-α production by tumor-associated plasmacytoid dendritic cells. Int. J. Cancer.

[29] Giorello M.B., Matas A., Marenco P., Davies K.M., Borzone F.R., de Luján Calcagno M., García-Rivello H., Wernicke A., Martinez L.M., Labovsky V. (2021). CD1a- and CD83-positive dendritic cells as prognostic markers of metastasis development in early breast cancer patients. Breast Cancer.

[30] Arantes Perez A., Balabram D., De Almeida Salles M., Gobbi H. (2014). Ductal Carcinoma In Situ of the Breast: Correlation between Histopathological Features and Age of Patients. Diagn. Pathol..

[31] Coventry B.J., Lee P.L., Gibbs D., Hart D. (2002). Dendritic cell density and activation status in human breast cancer-CD1a, CMRF-44, CMRF-56 and CD-83 expression. Br. J. Cancer.

[32] López C., Gibert-Ramos A., Bosch R., Korzynska A., García-Rojo M., Bueno G., García-Fontgivell J.F., Martínez-González S., Fontoura L., Gras Navarro A. (2021). Differences in the Immune Response of the Nonmetastatic Axillary Lymph Nodes between Triple-Negative and Luminal a Breast Cancer Surrogate Subtypes. Am. J. Pathol..

[33] Ozkan-Gurdal S., Cabioglu N., Ozcinar B., Muslumanoglu M., Ozmen V., Kecer M., Yavuz E., Igci A. (2014). Factors predicting microinvasion in ductal carcinoma in situ. Asian Pac. J. Cancer Prev..

[34] Fujii T., Yanai K., Tokuda S., Nakazawa Y., Kurozumi S., Obayashi S., Yajima R., Hirakata T., Kuwano H. (2017). Clinicopathological features of ductal carcinoma in situ from 18F-FDG-PET findings. Anticancer Res..

[35] al Nemer A.M. (2017). Histologic factors predicting invasion in patients with ductal carcinoma in situ (DCIS) in the preoperative core biopsy. Pathol. Res. Pract..

[36] Lejeune M., Reverté L., Sauras E., Gallardo N., Bosch R., Roso A., Petit A., Peg V., Riu F., García-Fontgivell J. (2023). Prognostic Implications of the Residual Tumor Microenvironment after Neoadjuvant Chemotherapy in Triple-Negative Breast Cancer Patients without Pathological Complete Response. Cancers.

[37] Argenziano M., Occhipinti S., Scomparin A., Angelini C., Novelli F., Soster M., Giovarelli M., Cavalli R. (2022). Exploring chi-tosan-shelled nanobubbles to improve HER2 + immunotherapy via dendritic cell targeting. Drug Deliv. Transl. Res..

[38] Kovats S. (2015). Estrogen receptors regulate innate immune cells and signaling pathways. Cell Immunol..

[39] Tabar L., Chen H.H.T., Yen M.F.A., Tot T., Tung T.H., Chen L.S., Chiu Y.H., Duffy S.W., Smith R.A. (2004). Mammographic tumor features can predict long-term outcomes reliably in women with 1-14-mm invasive breast carcinoma: Suggestions for the reconsideration of current therapeutic practice and the TNM classification system. Cancer.

[40] Valenzi E., Tabib T., Papazoglou A., Sembrat J., Trejo Bittar H.E., Rojas M., Lafyatis R. (2021). Disparate Interferon Signaling and Shared Aberrant Basaloid Cells in Single-Cell Profiling of Idiopathic Pulmonary Fibrosis and Systemic Sclerosis-Associated Interstitial Lung Disease. Front. Immunol..

[41] Kafaja S., Valera I., Divekar A.A., Saggar R., Abtin F., Furst D.E., Khanna D., Singh R.R. (2018). pDCs in lung and skin fibrosis in a bleomycin-induced model and patients with systemic sclerosis. JCI Insight.

[42] Peng D., Fu M., Wang M., Wei Y., Wei X. (2022). Targeting TGF-β signal transduction for fibrosis and cancer therapy. Mol. Cancer.

[43] Huang L., Rong Y., Tang X., Yi K., Qi P., Hou J., Liu W., He Y., Gao X., Yuan C. (2022). Engineered exosomes as an In Situ DC-primed vaccine to boost antitumor immunity in breast cancer. Mol. Cancer.

[44] Jugniot N., Dahl J.J., Paulmurugan R. (2022). Immunotheranostic microbubbles (iMBs)—A modular platform for dendritic cell vaccine delivery applied to breast cancer immunotherapy. J. Exp. Clin. Cancer Res..

[45] Bulgarelli J., Tazzari M., Granato A.M., Ridolfi L., Maiocchi S., de Rosa F., Petrini M., Pancisi E., Gentili G., Vergani B. (2019). Dendritic Cell Vaccination in Metastatic Melanoma Turns “Non-T Cell Inflamed” Into “T-Cell Inflamed” Tumors. Front. Immunol..

[46] Qian D., Li J., Huang M., Cui Q., Liu X., Sun K. (2023). Dendritic cell vaccines in breast cancer: Immune modulation and immunotherapy. Biomed. Pharm..

[47] Ridolfi L., de Rosa F., Fiammenghi L., Petrini M., Granato A.M., Ancarani V., Pancisi E., Soldati V., Cassan S., Bulgarelli J. (2018). Complementary vaccination protocol with dendritic cells pulsed with autologous tumour lysate in patients with resected stage III or IV mela-noma: Protocol for a phase II randomised trial (ACDC Adjuvant Trial). BMJ Open.

[48] Lester S.C., Bose S., Chen Y.-Y., Connolly J.L., de Baca M.E., Fitzgibbons P.L., Hayes D.F., Kleer C., O’Malley F.P., Page D.L. (2009). CAP Laboratory Improvement Programs Protocol for the Examination of Specimens from Patients with Invasive Carcinoma of the Breast. Arch. Pathol. Lab. Med..

