# Supplementary material for: Dendritic Cell Subpopulations Are Associated with Morphological Features of Breast Ductal Carcinoma In Situ
Source: Int J Mol Sci. 2023 Jun 8;24(12):9918. doi: 10.3390/ijms24129918 (PMC10298341; doi:10.3390/ijms24129918)
Supplement: Supplementary file 1 [file ijms-24-09918-s001.zip › ijms-2422153-supplementary.pdf]

Table S1. Relationships between number of cells (*N*) of investigated DC subpopulations and different histological features in DCIS. DC numbers are expressed as median and interquartile range (Me [Q1-Q3]). Number of CD1a+ cells is divided by the number of DCIS-occupied grid fields.

	Solid			Cribriform			Micropapillary			Papillary			Comedo-type			Apocrine		
	Absent	Present	p/p^{BH}	Absent	Present	p/p^{BH}	Absent	Present	p/p^{BH}	Absent	Present	p/p^{BH}	Absent	Present	p/p^{BH}	Absent	Present	p/p^{BH}
**CD1a+ intratumoral**	0.4 [0.0-3.6]	1.3 [0.3-2.4]	0.5/-	1.3 [0.1-2.0]	1.4 [0.1-2.8]	0.8/-	1.2 [0.1-2.3]	1.6 [0.1-3.5]	0.4/-	1.3 [0.1-2.6]	1.4 [0.2-2.7]	0.9/-	1.2 [0.1-2.7]	1.7 [0.3-2.3]	0.5/-	1.3 [0.1-2.4]	1.6 [0.1-4.7]	0.5/-
**CD1a+ peritumoral**	0 [0-30]	10 [2-91]	0.013/ 0.08	21 [2-130]	3 [0-54]	0.042/ 0.2	6 [0-81]	5 [0-102]	0.9/-	6 [0-84]	6 [0-79]	0.6/-	5 [0-76]	10 [2-155]	0.3/-	5 [0-83]	13 [0-201]	0.7/-
**CD123+ peritumoral**	46 [15-131]	97 [52-204]	0.03/ 0.1	111 [59-161]	62 [22-199]	0.08/-	97 [53-161]	57 [20-199]	0.2/-	101 [46-184]	61 [15-118]	0.08/-	80 [24-152]	118 [104-208]	0.052/-	84 [31-154]	134 [111-222]	0.2/-
**DC-SIGN+ peritumoral**	31 [13-47]	55.5 [24-104]	0.032/ 0.4	52 [18-105]	45 [22-76]	0.6/-	46 [24-84]	45 [16-104]	1.0/-	46 [16-102]	45 [22-72]	0.9/-	45 [21-77]	104 [21-141]	0.1/-	46 [21-90]	31 [8-124]	1.0/-
**DC-LAMP+ peritumoral**	62 [2-107]	74.0 [16-167]	0.08/-	123 [65-176]	30 [4-107]	**0.002/ 0.009**	72 [14-161]	74 [4-166]	0.8/-	79 [14-167]	41 [6-92]	0.1/-	61 [6-119]	177 [154-351]	**<0.001/ <0.001**	66 [7-156]	107 [98-248]	0.2/-

	Comedo-necrosis			Ductal spread			Lobular cancerization			Paget disease			Microcalcification			Microinvasion		
	Absent	Present	p/p^{BH}	Absent	Present	p/p^{BH}	Absent	Present	p/p^{BH}	Absent	Present	p/p^{BH}	Absent	Present	p/p^{BH}	Absent	Present	p/p^{BH}
**CD1a+ intratumoral**	1.7 [0.1-4.0]	1.2 [0.1-2.1]	0.2/-	0.3 [0.0-1.6]	1.4 [0.3-2.7]	0.033/ 0.2	1.0 [0.1-3.0]	1.4 [0.3-2.5]	0.6/-	1.2 [0.1-2.3]	3.2 [1.3-6.3]	0.005/ 0.06	0.3 [0.0-2.8]	1.3 [0.3-2.5]	0.3/	1.3 [0.1-2.6]	1.1 [0.1-3.2]	1.0/-
**CD1a+ peritumoral**	11 [1-91]	4 [0-80]	0.4/-	3 [0-30]	10 [0-102]	0.1/-	6 [0-83]	5 [0-86]	0.8/-	4 [0-57]	218 [91-311]	**<0.001/ <0.001**	8 [0-166]	6 [0-82]	0.8/-	6 [0-83]	5 [0-125]	0.9/-
**CD123+ peritumoral**	58 [15-123]	110 [49-210]	0.02/ 0.1	51 [15-85]	107 [52-203]	0.006/ 0.07	62 [23-131]	123 [54-204]	0.03/ 0.1	84 [26-152]	155 [100-261]	0.049/ 0.1	74 [28-176]	93 [36-169]	0.5/-	86 [34-176]	96 [24-154]	1.0/-
**DC-SIGN+ peritumoral**	45 [27-74]	48 [18-103]	0.7/-	43 [31-54]	50 [16-104]	0.6/-	44 [30-82]	48 [11-109]	0.8/-	44 [21-82]	105 [62-152]	0.06/-	54 [15-147]	43 [21-95]	0.5/-	45 [21-86]	64 [15-104]	0.5/-
**DC-LAMP+ peritumoral**	28 [6-103]	87 [24-177]	**0.007/ 0.02**	7 [2-18]	107 [45-172]	**<0.001/ <0.001**	37 [3-119]	107 [48-193]	**0.002/ 0.009**	66 [6-161]	147 [119-166]	0.044/ 0.09	53 [8-113]	77 [10-166]	0.3/-	66 [7-161]	84 [72-165]	0.3/-

Abbreviations: CD1a—cluster of differentiation 1a, CD123—cluster of differentiation 123, DC—dendritic cell, DCIS—ductal carcinoma in situ, DC-LAMP—dendritic cell lysosome-associated membrane glycoprotein, DC-SIGN—dendritic cell-specific intercellular adhesion molecule-3-grabbing non-integrin, p/p^{BH}—p-value and p-value after Benjamini-Hochberg correction (respectively).

Table S2. Differences in densities of DCs subset infiltrate in DCIS tumors of various nuclear grades. DC numbers are expressed as median and interquartile range (Me [Q1-Q3]). Number of $CD1a^+$ cells is divided by the number of DCIS-occupied grid fields.

Nuclear grade	$CD1a^+$ intratumoral cell number$^\$$ [N]	p- value	$CD1a^+$ peritumoral cell number [N]	p-value	$CD123^+$ peritumoral cell number [N]	p-value	$DC\text{-}SIGN^+$ peritumoral cell number [N]	p-value	$DC\text{-}LAMP^+$ peritumoral cell number [N]	p value
1	0.7 [0-0.2]		0 [0-3]*		37 [8-75]*,#		38 [15-54]		7 [3-13]*	
2	1.4 [0.1-3]	0.083	4 [0-57]*	0.021	58 [22-134]*	<0.001	42 [22-74]	0.108-	46 [5-87]#	<0.001
3	1.2 [0.3-2.1]		50 [1-163]		143 [101-282]#		83 [21-136]		160 [78-242]*,#	

ANOVA Kruskal–Wallis test was performed. DCs numbers are expressed as median and interquartile range (Me [Q1-Q3]). Symbol ($) indicates that the number of $CD1a^+$ DCs was averaged per number of 3x3 grid fields (field size: 1023x767.5 μm/1637x1228 pixels) occupied by DCIS foci. Symbols (*, #) indicate significant differences between given groups found via post-hoc multiple comparison of average ranks. **Abbreviations:** CD1a—cluster of differentiation 1a, CD123—cluster of differentiation 123, DC—dendritic cell, DCIS—ductal carcinoma in situ, DC-LAMP—dendritic cell lysosome-associated membrane glycoprotein, DC-SIGN—dendritic cell-specific intercellular adhesion molecule-3-grabbing non-integrin.

Table S3. Correlations between number of cells (*N*) of investigated DC subpopulations and nuclear receptors expression or maximal tumor size (histological, ultrasonographic or mammographic) of DCIS. The Benjamini-Hochberg correction was applied.

	ER (%)		PR (%)		Maximal tumor size in histology (mm)		Maximal tumor size in ultrasonography (mm)		Maximal tumor size in mammography (mm)	
	**Spearman R**	p/p^{BH}	**Spearman R**	p/p^{BH}	**Spearman R**	p/p^{BH}	**Spearman R**	p/p^{BH}	**Spearman R**	p/p^{BH}
**CD1a+ intratumoral**	-0.16	0.2/-	-0.20	0.074/-	0.19	0.074/-	0.14	0.4/-	0.38	0.022/0.1
**CD1a+ peritumoral**	-0.38	**<0.001/0.002**	-0.43	**<0.001/<0.001**	0.20	0.058/-	0.13	0.5/-	0.29	0.089/-
**CD123+ peritumoral**	-0.43	**<0.001/<0.001**	-0.48	**<0.001/<0.001**	0.29	**0.007/0.011**	0.26	0.1/-	0.31	0.069/-
**DC-SIGN+ peritumoral**	-0.17	0.2/-	-0.17	0.1/-	0.24	0.024/0.1	0.05	0.8/-	0.31	0.064/-
**DC-LAMP+ peritumoral**	-0.62	**<0.001/<0.001**	-0.59	**<0.001/<0.001**	0.42	**<0.001/<0.001**	0.25	0.2/-	0.32	0.060/-

Abbreviations: CD1a—cluster of differentiation 1a, CD123—cluster of differentiation 123, DC—dendritic cell, DCIS—ductal carcinoma in situ, DC-LAMP—dendritic cell lysosome-associated membrane glycoprotein, DC-SIGN—dendritic cell-specific intercellular adhesion molecule-3-grabbing non-integrin, ER—estrogen receptor, PR—progesterone receptor, p/p^{BH}—p-value and p-value after Benjamini-Hochberg correction (respectively).
